# Effect of Austenitising Temperature on Mechanical Properties of Nanostructured Bainitic Steel

**DOI:** 10.3390/ma10080874

**Published:** 2017-07-28

**Authors:** Jing Zhao, Jiemin Li, Honghong Ji, Tiansheng Wang

**Affiliations:** 1 State Key Laboratory of Metastable Materials Science and Technology, Yanshan University, Qinhuangdao 066004, China; zjjysu@163.com (J.Z.); lijiemin01@163.com (J.L.); jhh123ykl@163.com (H.J.); 2 National Engineering Research Center for Equipment and Technology of Cold Strip Rolling, Yanshan University, Qinhuangdao 066004, China

**Keywords:** steel, phase transformation, nanostructured bainite, mechanical property

## Abstract

Nanostructured bainite was obtained in high-carbon Si-Al-rich steel by low-temperature (220–260 °C) isothermal transformation after austenitisation at different temperatures (900 °C, 1000 °C, and 1150 °C). Improved strength-ductility-toughness balance was achieved in the nanostructured bainitic steel austenitised at low temperatures (900 °C and 1000 °C). Increasing the austenitising temperature not only coarsened prior austenite grains and bainite packets, but also increased the size and fraction of blocky retained austenite. High austenitising temperature (1150 °C) remarkably decreased ductility and impact toughness, but had a small effect on strength and hardness.

## 1. Introduction

Novel nanostructured bainitic steels are designed to form nanostructures consisting of 20–40-nm-thick plates of bainitic ferrite and retained austenite by low-temperature isothermal bainite transformation [1,2,3]. The nanostructure presents an excellent combination of strength (ultimate tensile strength (UTS) of ~2.5 GPa) and toughness (up to 40 MPa·m^1/2^) [4,5]. These nanostructured bainitic steels are typically composed of high C, high Si, and a mass of austenite stabilizer (such as Mn and Cr) to ensure low-temperature isothermal bainite transformation without carbide precipitation. This process requires an isothermal transformation time as long as several to tens of days. Expensive elements such as Co or Co + Al are added in steel to accelerate bainitic transformation [6,7]. Nanostructured bainitic steel with high hardness is developed by increasing C content, decreasing Cr content, and removing Mn [7]. Moreover, bainitic transformation is accelerated by reducing C, Mn, Cr, and Mo contents and by refining prior austenite grain size with Nb addition [8,9]. This process can also be accelerated by increasing Si content to 3 wt % and reducing Mn and Cr contents [10], instead of adding Co and Al. In addition, Huang et al. [11] proposed a new low-Mn high-Cr nanostructured bainitic steel with increased hardness. The formation of low-temperature bainite was effectively promoted by considerably reducing Mn content and adding a small amount of Al. Most studies are focused on alloy design and the effect of heat treatment—in particular, isothermal transformation temperature—on microstructure and mechanical properties of nanostructured bainitic steels. Xu et al. [12] recently reported that higher austenitisation temperature can remarkably accelerate isothermal bainitic transformation in medium-C superbainite steel. Wu et al. [13] and Kong et al. [14] revealed that coarse austenite grain size obtained by austenitising at high temperature can accelerate isothermal bainitic transformation of high-C superbainitic steel. Wu et al. [13] also found that coarse austenite grain increases the width and length of bainitic ferrite sheaves in high-C superbainitic steel. Hence, mechanical properties are inevitably affected by austenitising temperature. This work was performed to evaluate the effect of austenitising temperature on microstructure and mechanical properties of high-C nanostructured bainitic steel.

## 2. Experimental Procedure

Steel was smelted in a vacuum induction furnace and cast into a 170-mm diameter ingot with chemical composition of 0.80C–0.81Cr–1.59Si–1.35Mn–0.89W–1.47Al–0.01P–0.005S (wt %). The ingot was heated to 1220 °C for 4 h and hot rolled into a 20-mm-thick plate with finish rolling temperature of approximately 880 °C. The hot-rolled plate was spheroidised by heating the plate to 810 °C for 100 min and cooling in a furnace to 720 °C for 5 h. The plate was then air-cooled to room temperature. The *A_c_*_1_ and *A_ccm_* temperature of the steel were 726 °C and 823 °C. The martensite start temperature (*M_s_*) was 180 °C and was determined by dilatometry on a Gleeble-3500 thermomechanical simulator. The specimens were cut into square bars (11 mm × 11 mm × 55 mm) and plates (130 mm × 20 mm × 5 mm) by wire electrode discharging. The longitudinal and thick directions were parallel to the rolling and normal directions, respectively. The square bars and plates were isothermally transformed at 220 °C, 240 °C, and 260 °C for 24, 12, and 4 h, respectively, in molten salt composed of sodium nitrite and potassium nitrate (1:1 in weight) after the specimens were austenitised for 30 min at 900 °C, 1000 °C, and 1150 °C in a muffle furnace. Austenitisation at different temperatures was designed to alter the size of prior austenite grain. The isothermally transformed square bars were machined into Charpy impact samples with dimensions of 10 mm × 10 mm × 55 mm and a U-shaped notch with size of 2 mm in width and 2 mm in depth to evaluate the room-temperature impact toughness. The isothermally transformed plates were machined into tensile samples with gauge size of 30 mm × 10 mm × 2.8 mm to evaluate the room-temperature tensile properties on a MTS Landmark Servohydraulic Test System at a cross head moving rate of 2 mm·min^−1^. Vickers hardness was measured on a hardometer (FM-ARS 9000) at a load of 0.5 kgf. Impact fracture surface morphologies were examined using a scanning electron microscope (SEM, Hitachi S-4800, Hitachi, Tokyo, Japan).

Microstructures and prior austenite grain boundaries in isothermally transformed samples were examined using an optical microscope (OM, Axiover 200MAT, Zeiss, Heidenheim, Germany) and a transmission electron microscope (TEM, JEM-2010, JEOL, Musashino, Japan). The volume fraction of retained austenite was measured by X-ray diffraction (XRD, D/max-2500/PC, Rigaku, Tokyo, Japan) according to the method described in [15]. Samples for OM and XRD examinations were ground to ensure the removal of oxidation and decarburisation layers. Then, the samples were mechanically polished and chemically etched using 4% nital. Prior austenite grain boundaries were displayed using an etchant composed of picric acid (1.4 g), sodium dodecyl benzene, sulphonate (2 g), and distilled water (50 mL) by heating to ~70 °C in a water bath for 2–3 min. The mean value and standard deviation of prior austenite grain size were obtained by statistical analysis using diameters of more than 100 grains in over five OM viewing fields. Foils for TEM were sliced into ~0.5 mm thickness by electro-discharging, and the foils were mechanically ground to ~50 μm in thickness using a waterproof abrasive paper. The foils were then thinned to perforation on a TenuPol-5 twin-jet electropolishing device using an electrolyte composed of 7 vol % perchloric acid and 93 vol % glacial acetic acid at room temperature.

## 3. Results and Discussion

### 3.1. Microstructure

**Figure 1:**
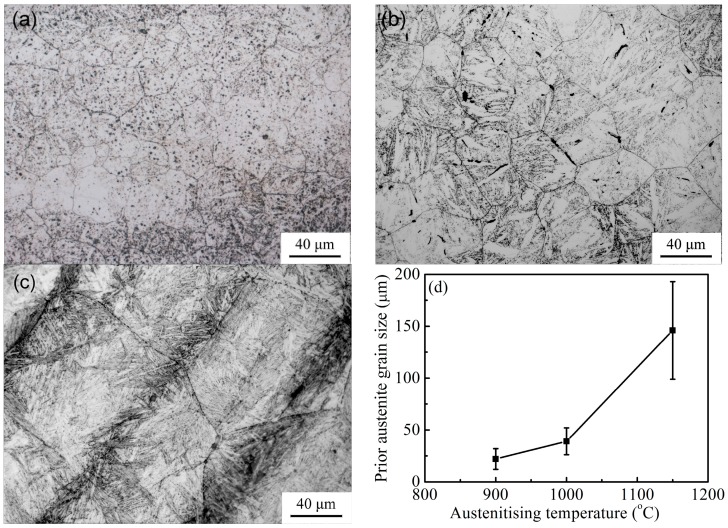
Optical microscopy (OM) images of prior austenite grains in samples austenitised at (**a**) 900 °C; (**b**) 1000 °C; (**c**) 1150 °C; and (**d**) dependence of prior austenite grain size on austenitising temperature.

Figure 1a–c show the typical OM images of prior austenite grains in samples austenitised at 900 °C, 1000 °C, and 1150 °C, respectively. The dependence of prior austenite grain on austenitising temperature is demonstrated in Figure 1d. The mean size of prior austenite grains increased with austenitising temperature, and the grain size became extremely large as austenitising temperature reached 1150 °C. 

**Figure 2:**
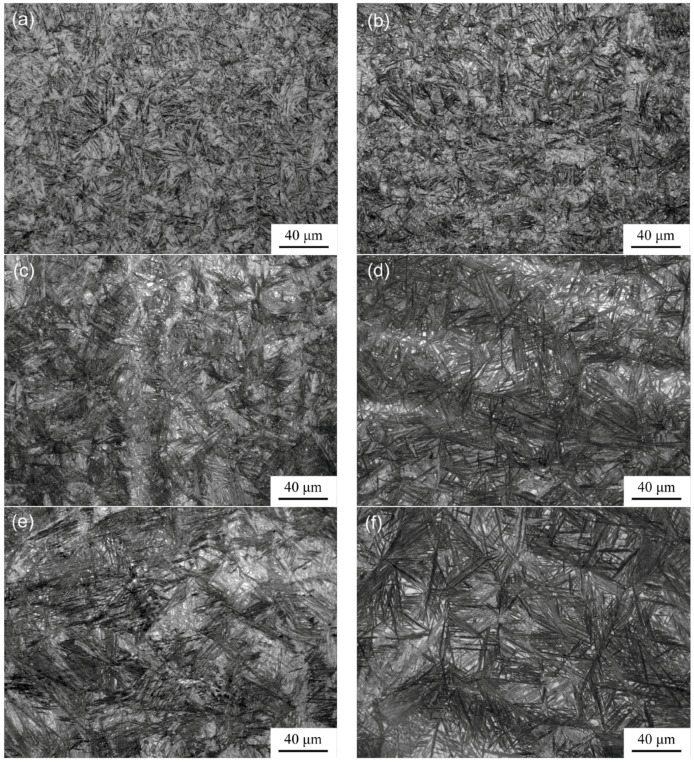
OM images of microstructures in samples isothermally transformed at (**a**,**c**,**e**) 220 °C and (**b**,**d**,**f**) 260 °C after austenitised at (**a**,**b**) 900 °C; (**c**,**d**) 1000 °C; and (**e**,**f**) 1150 °C.

Figure 2 reveals typical OM microstructures of samples isothermally transformed at 220 °C and 260 °C at austenitising temperatures of 900 °C, 1000 °C, and 1150 °C. The black or dark grey needles are bainite, and white blocks are retained austenite in all isothermally transformed samples. No undissolved carbides are observed, and this is consistent with the results of XRD patterns. The packet size and needle length were measured by Image-Pro Plus software. The size of the packet in Figure 2a–e is ~7 ± 1, 9 ± 2, 14 ± 2, 18 ± 4, 24 ± 6, and 27± 9 μm, respectively. The length of the needle in Figure 2a–e is ~11 ± 2, 18 ± 4, 26 ± 5, 29 ± 7, 41 ± 12, and 42 ± 14 μm, respectively. The packet size and needle length—rather than thickness—of bainite markedly increased with austenitising temperature. Increasing the isothermal transformation temperature mainly resulted in increased thickness of bainite needles, rather than in the packet size and the needle length. This phenomenon is attributed to the fact that the austenite grain size is greater at high austenitising temperature. This characteristic reduces the density of nucleation sites of bainitic ferrite, increasing the packet size and the needle length. As we all know, at lower isothermal transformation temperature, the transformation driving force or degree of supercooling is larger, thereby increasing the nucleation rate of bainite and thinning the bainite needles. The fraction of blocky retained austenite was measured by the Image-Pro Plus software, and that in Figure 2a–e is ~8.3%, 9.1%, 11.1%, 13.3%, 12.5%, and 15.4%, respectively. Therefore, the size and fraction of blocky retained austenite increases with isothermal transformation and austenitising temperatures (Figure 2). Higher austenitising temperature leads to increased blocky austenite [16]. Retained austenite occurs in two forms in nanostructured bainitic steels. The blocky austenite is trapped between different bainite packets, and film-like austenite is trapped between bainitic ferrite laths.

**Figure 3:**
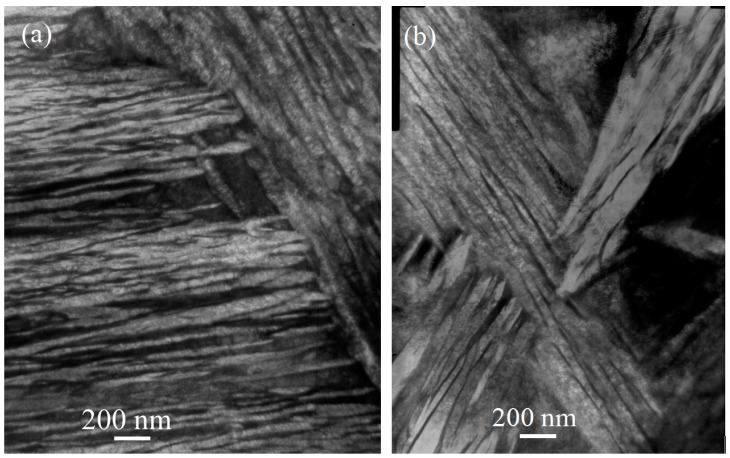
TEM images of microstructures in samples isothermally transformed at (**a**) 220 °C; and (**b**) 260 °C after austenitised at 1000 °C.

TEM observations were performed to examine the austenite films and bainitic ferrite laths. Figure 3 illustrates the transmission electron micrographs of the microstructures in the samples isothermally transformed at 220 °C and 260 °C after austenitisation at 1000 °C. The bright laths are bainitic ferrite, and the dark laths are retained austenite. Bainitic ferrite laths and austenite films exist within bainite packets. The mean thickness (*t*) of the bainite laths was obtained by measuring the mean lineal intercept *L_T_* in a direction normal to the lath length and by stereologically correcting in terms of *L_T_* = *πt/2* [17]. The lath thicknesses of bainitic ferrite was 34 ± 10 and 40 ± 9 nm in the samples isothermally transformed at 220 °C and 260 °C, and those are less than 100 nm. Higher isothermal temperature generated thicker bainitic ferrite laths. No undissolved and precipitated carbides were observed upon extensive TEM examinations, which suggests that nanostructured bainite was formed in the isothermally transformed samples. 

**Figure 4:**
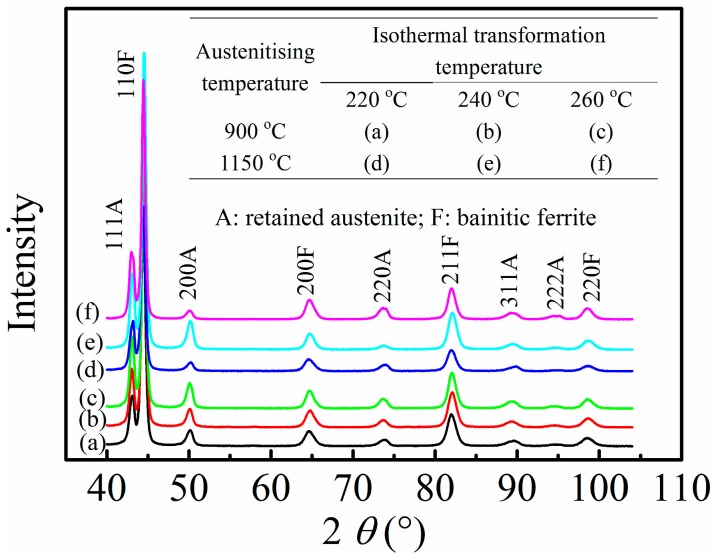
XRD patterns of samples isothermally transformed at 220 °C, 240 °C, and 260 °C after austenitised at (**a**–**c**) 900 °C; and (**d**–**f**) 1150 °C.

Figure 4 shows XRD patterns of samples isothermally transformed at 220 °C, 240 °C, and 260 °C after austenitisation at 900 °C and 1150 °C. All isothermally transformed samples were composed of bainitic ferrite with body-centered cubic structure, and retained austenite was with face-centred cubic structure. No carbide diffraction peaks were found in the XRD patterns. The diffraction angles of reflections with similar indices decreased for retained austenite, but increased for bainitic ferrite, with increasing isothermal transformation temperature. This phenomenon suggests that elevating isothermal transformation temperature can increase the C content of retained austenite and decrease the C content of bainitic ferrite. However, austenitising temperature had little effect on diffraction angle. Using reflections 111, 200, 220, and 311 of austenite, as well as 110, 200, 211, and 220 of bainitic ferrite, the volume fractions of retained austenite in samples isothermally transformed at 220 °C, 240 °C, and 260 °C were calculated to be approximately 26%, 29%, and 31%, respectively, for austenitisation at 900 °C. The corresponding volume fractions were 28%, 29%, and 29% for austenitisation at 1150 °C. The fraction of retained austenite evaluated via XRD is the total of the fractions of austenite films and blocks. The retained austenite fraction appeared to have a small difference in isothermal transformation temperature and austenitising temperature did not significantly affect the fraction of retained austenite that is limited by the *T*_0_’ curve. This phenomenon implies the absence of undissolved or precipitated carbides in isothermally transformed samples. The reduction of C in austenite accelerates bainite transformation because of the decrease in austenite stability. The absence of carbides resulted in similar contents of C and other alloy elements in austenite from 900 to 1150 °C. Therefore, similar fractions of retained austenite were obtained in samples isothermally transformed at identical temperatures after austenitisation at different temperatures. 

### 3.2. Mechanical Properties

**Figure 5:**
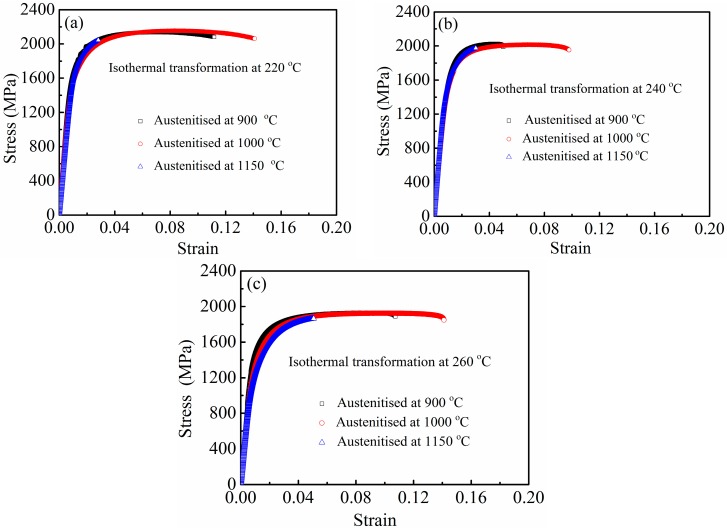
Engineering stress-strain curves of samples isothermally transformed at (**a**) 220 °C; (**b**) 240 °C; and (**c**) 260 °C after austenitisation at different temperatures.

Engineering stress-strain curves of samples isothermally transformed at 220 °C, 240 °C, and 260 °C after austenitisation at different temperatures are presented in Figure 5. Continuous yielding occurred during tensile deformation. This phenomenon is attributed to high-density dislocations in bainitic ferrite and a large amount of retained austenite that has higher work hardening capacity induced by dislocation multiplication and strain-induced martensite. Continuous yielding was observed in the other two nanostructured bainitic steels [18]. The mechanical properties—namely, UTS, yield strength (YS, 0.2% proof stress), total elongation, hardness, and impact toughness—are given in Table 1. Enhanced balance in strength-ductility-toughness was achieved in samples isothermally transformed at given temperatures and austenitised at 900 °C and 1000 °C. However, high austenitising temperature (1150 °C) reduced ductility and toughness. This phenomenon may have been caused by the following factors. Higher austenitising temperature results in a greater amount and larger size of blocky retained austenite that readily transforms into brittle martensite because of the applied stress or strain during tensile or impact test. This characteristic cannot effectively blunt the crack. Moreover, high austenitising temperature coarsens prior austenite grains and bainite packets. Garbarz et al. [19] showed that impact toughness of nanostructured bainite-austenite steel can be improved by introducing a small amount of martensite plates before isothermal transformation to divide prior austenite grains and refine bainite packets.

**Figure 6:**
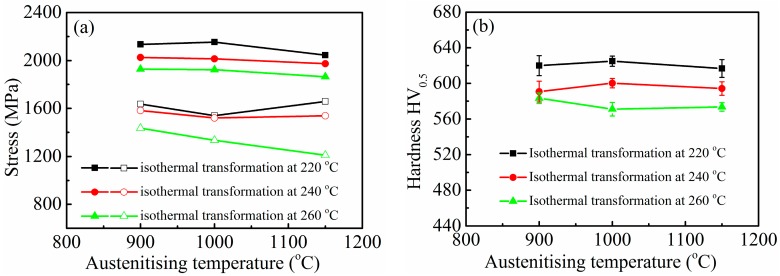

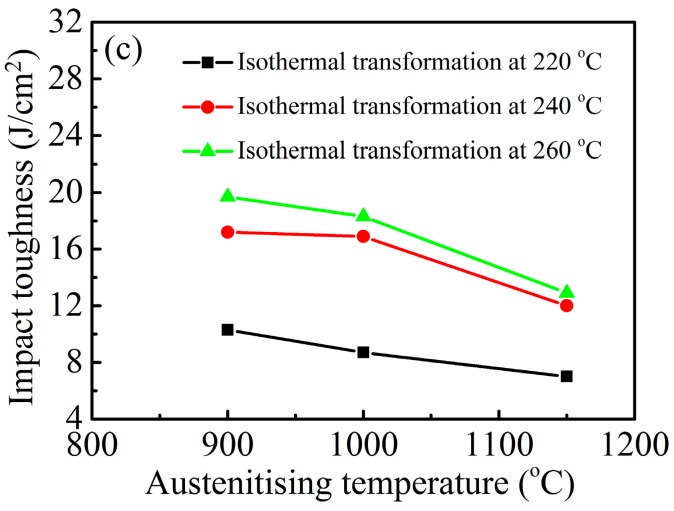
(**a**) Strength; (**b**) hardness; and (**c**) impact toughness as a function of austenitising temperature for isothermally transformed samples. Note that tensile strength and yield strength are represented in solid and open symbols, respectively.

Figure 6 shows the effect of austenitising temperature on strength, hardness, and impact toughness of samples isothermally transformed at different temperatures. An indistinctive change in the strength and hardness was caused by increasing austenitising temperature, whereas a comparatively marked decrease was caused by increasing isothermal transformation temperature (Figure 6a,b). The strength and hardness of nanostructured bainitic steels are affected by many factors, such as thickness of bainitic ferrite laths and retained austenite films, fractions of bainitic ferrite and blocky and film-like retained austenite, C contents in bainitic ferrite and blocky and film-like retained austenite, mechanical stability of retained austenite, size of blocky retained austenite size, and dislocation densities in bainitic ferrite and retained austenite. The interaction among these factors inevitably results in complex effects on strength and hardness. Reference [16] reported that the mean free path for slip is related to the bainitic plate thickness, rather than plate length. This characteristic suggests that the major microstructural contributor to strength is the fine sub-unit size, rather than the sheaf or austenite grain size. Avishan et al. [18] also discussed the good linear relation between strength (YS and UTS) and the ratio of the volume fraction to the thickness of the bainitic ferrite, such that both YS and UTS increase with the aforementioned ratio. Lowering isothermal transformation temperature not only reduces bainitic lath thickness and increases the bainitic ferrite fraction, but also increases the dislocation density and C content in bainitic ferrite. These phenomena consequently enhance strength. In addition, decreasing isothermal transformation temperature reduces impact toughness (Figure 6c). Elevating isothermal transformation temperature increases the C content and fraction of retained austenite and decreases C content of bainitic ferrite. This phenomenon reduces the difference in strength between bainitic ferrite and retained austenite, consequently improving impact toughness. However, high isothermal transformation temperature increases the fraction of blocky retained austenite, and this characteristic is detrimental to impact toughness. 

**Figure 7:**
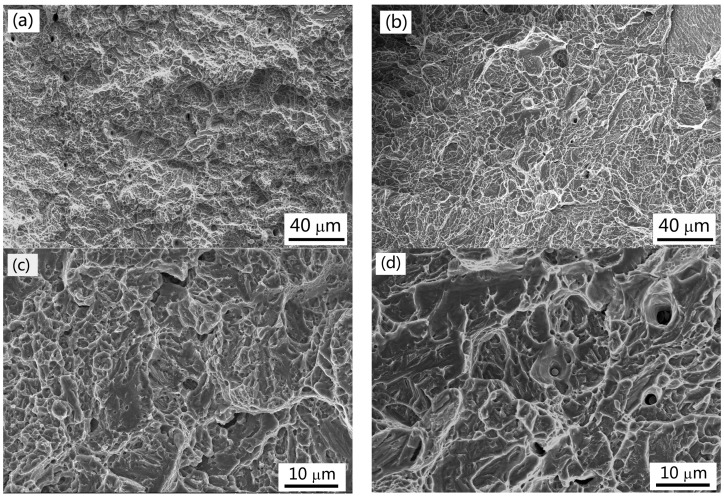
SEM fractographs of impact samples isothermally transformed at 260 °C after austenitised at (**a**,**c**) 900 °C and (**b**,**d**) 1150 °C. (**a**,**b**) are low magnification images; (**c**,**d**) are high magnification images.

Figure 7 shows the SEM fractographs of impact samples isothermally transformed at 260 °C after austenitisation at 900 °C and 1150 °C. The fracture morphologies display cleavage facets, tearing ridges, and ductile dimples, suggesting that the fracture is in a mixed mode of ductile and brittle phases. The low-magnification SEM fractographs in Figure 7a,b show that the fracture surface roughness of sample austenitised at 1150 °C is less than that of the sample austenitised at 900 °C. Additionally, the high-magnification SEM fractographs in Figure 7c,d illustrate that cleavage facet size in the fracture surface of sample austenitised at 1150 °C is larger than that of sample austenitised at 900 °C. Therefore, high austenitising temperature leads to low impact toughness.

## 4. Conclusions

Nanostructured bainite microstructure with ultrahigh strength (UTS 1865–2154 MPa) and hardness (571–625 HV_0.5_) was obtained in high-carbon Si-Al-rich steel by low-temperature (220–260 °C) isothermal transformation after austenitisation at 900 °C, 1000 °C, and 1150 °C. Optimum balance in strength-ductility-toughness was achieved at low austenitisation temperatures (900 °C and 1000 °C). Increasing the austenitising temperature not only coarsened prior austenite grains and bainite packets, but also increased the size and fraction of blocky retained austenite. Austenitising temperature had an indistinctive effect on strength and hardness. However, austenitising at 1150 °C resulted in a remarkable decrease in ductility and impact toughness because extremely large austenite grains form at this austenitising temperature.

## Figures and Tables

**Table 1:** Results of mechanical properties of samples isothermally transformed. TEL: total elongation; UTS: ultimate tensile strength; YS: yield strength.

Austenitising Temperature (°C)	Isothermal Temperature (°C)	Hardness (HV_0.5_)	UTS (MPa)	YS (MPa)	TEL (%)	Impact Toughness (J/cm^2^)
900	220	619.9 ± 11.2	2135	1636	10.0	10.3
	240	590.7 ± 11.7	2026	1582	4.0	17.2
	260	583.3 ± 6.0	1929	1435	10.0	19.7
1000	220	624.9 ± 5.8	2154	1540	13.0	8.7
	240	600.2 ± 5.3	2015	1520	8.6	16.9
	260	571.0 ± 7.7	1924	1334	12.9	18.3
1150	220	616.7 ± 10.0	2045	1658	1.8	7.0
	240	594.2 ± 7.6	1974	1538	2.1	12.0
	260	573.5 ± 4.8	1865	1210	4.0	12.9
